# Consolidation of Medical Colleges

**Published:** 1878-10

**Authors:** 


Consolidation of Medical Colleges.—At the last meeting of the Ohio State Medical Society a committee was appointed on the "consolidation of medical colleges." The idea was that too many colleges existed in Ohio, and that the profession would be benefited by concentrating the present teaching material into a smaller compass. The committee consists of one member from each of the seven faculties of the seven regular medical colleges of the State. The idea is a capital one if well developed.—*Detroit Lancet.*

